# Circulating levels of FAM19A5 are inversely associated with subclinical atherosclerosis in non-alcoholic fatty liver disease

**DOI:** 10.1186/s12902-021-00820-8

**Published:** 2021-08-03

**Authors:** Fatemeh Ali Yari, Parisa Shabani, Sara Karami, Negar Sarmadi, Hossein Poustchi, Ahmad Reza Bandegi

**Affiliations:** 1 Department of Biochemistry, School of Medicine, Semnan University of Medical Sciences, Semnan, Iran; 2 Liver and Pancreatobiliary Diseases Research Center, Digestive Diseases Research Institute, Tehran University of Medical Sciences, Tehran, Iran; 3 Digestive Diseases Research Center, Shariati Hospital, Tehran University of Medical Sciences, Tehran, Iran; 4 Research Center of Physiology, Semnan University of Medical Sciences, Semnan, Iran

**Keywords:** Nonalcoholic fatty liver disease, FAM19A5, Carotid artery intima-media thickness, Liver stiffness

## Abstract

**Background:**

Family with sequence similarity 19 (chemokine (C-C motif)-like) member A5 (FAM19A5) is a newly identified adipokine. There is a limited number of studies linking FAM19A5 to metabolic disorders. In the current study, we aimed to explore if FAM19A5 is associated with nonalcoholic fatty liver disease (NAFLD). We also sought to determine the possibility of FAM19A5 association with subclinical atherosclerosis in NAFLD patients.

**Methods:**

A total of 69 subjects including 37 NAFLD and 32 control subjects were included in this cross-sectional study. Plasma concentration of FAM19A5 was measured with the ELISA method. Carotid artery intima-media thickness (cIMT) was assessed by the ultrasonography.

**Results:**

Plasma concentration of FAM19A5 in patients with NAFLD was significantly lower in NAFLD patients than controls. Moreover, we observed significant negative correlations between plasma level of FAM19A5 and body mass index (BMI), visceral fat, alanine amino transferase (ALT), aspartate amino transferase (AST), liver stiffness (LS), and cIMT. Following stepwise multiple linear regression analysis, ALT and cIMT were the only determinants of FAM19A5 level.

**Conclusions:**

This is the first report to describe association of circulating FAM19A5 levels with NAFLD. Our findings provide further evidence showing relation of FAM19A5 with the risk of atherosclerosis. However, more studies are necessary to unravel the contribution of lower FAM19A5 levels to the NAFLD pathogenesis and the higher risk of atherosclerosis in these patients.

## Background

Non-alcoholic fatty liver disease (NAFLD) is the most common chronic liver disease with the prevalence of 25% in the general population [1]. NAFLD is closely associated with obesity and is regarded as the hepatic component of metabolic syndrome [2]. As one of the largest endocrine organs in the body, adipose tissue secrets a host of bioactive molecules, called adipokines [3]. Adipokines possess a wide spectrum of metabolic effects in the physiological and pathological processes [3]. Previous studies have reported the important roles of adipokines in the pathogenesis of NAFLD [4–7].

Family with sequence similarity 19 (chemokine (C-C motif)-like) member A5 (FAM19A5) belongs to a family with sequence similarity 19, which is composed of 5 highly homologous genes (FAM19A1–5). FAM19A5 is a secretory protein which is predominantly expressed in the brain and adipose tissues. It is first identified as an adipokine by Wang et al. [8]. They studied FAM19A5 expression in the epididymal adipose tissues of high-fat diet-induced obese and leptin receptor-deficient (db/db) mice. They observed reduced expression of FAM19A5 expression of the epididymal adipose tissues of obese mice [8]. In a recent clinical study, it has been indicated that circulating level of FAM19A5 is different in diabetic patients compared to controls. They also found association of FAM19A5 with the obesity and diabetes indicators [9].

Growing epidemiological evidence demonstrates association of NAFLD with subclinical atherosclerosis and increased prevalence of cardiovascular diseases [10–12]. Subclinical atherosclerosis is an early indicator of atherosclerotic risk which can predict future cardiovascular events. Subclinical atherosclerosis can be conveniently measured by carotid intima-media thickness (CIMT) [13–15]. Now NAFLD is considered as a potential independent risk factor for cardiovascular diseases [16]. Several studies reported greater carotid artery intimal medial thickness in ultrasound- diagnosed NAFLD patients [17–20]. Moreover, it has been revealed that adipokines contribute to the cardiovascular homeostasis which affects vessel remodeling [21]. Wang et al. showed FAM19A5 inhibits the vascular smooth muscle cells proliferation and ameliorates neointima formation in the injured rat carotid arteries [8]. Although several studies addressed contribution of FAM19A5 to neurological disorders [22–26], existing data on a relevant association of FAM19A5 and cardiac and metabolic disorders are very limited [8, 9]. To our knowledge, this is the first clinical observation pointing to the association of FAM19A5 with the pathogenesis of NAFLD.

The aim of this study was to compare the circulating levels of FAM19A5 in patients with NAFLD and controls and to investigate a potential association of FAM19A5 with fatty liver and atherosclerosis indices.

## Methods

### Study design and participants

A total of 69 subjects including 37 NAFLD and 32 control subjects were selected from Golestan Cohort Study [27]. This study was approved by the medical ethics committee of Semnan University of Medical Sciences, and the written informed consent was obtained from all participants according to the guidelines of Declaration of Helsinki. The diagnosis of NAFLD was established using ultasonography and fibroscan. All participants were male, aged 50–81 years old. The subjects were excluded if they had excessive alcohol consumption (> 30 g/d), diabetes, viral hepatitis, autoimmune liver disease, hemochromatosis, Wilson’s disease. None of the patients were taking medication that has been reported to cause steatosis.

### Anthropometric and laboratory evaluation

Anthropometric parameters including age, height, weight, blood pressure (BP), waist circumference (WC) were measured in accordance with the standardized protocols. Body mass index (BMI) was calculated as body weight (kg) divided by the square of height (m2). WC was measured at the midpoint between the lowest rib and the iliac crest.

Plasma samples were collected from participants following an overnight fasting and stored at − 80 °C until analysis. Fasting blood glucose (FBG), serum total cholesterol (TC), triglycerides (TG), high-density lipoprotein cholesterol (HDL-C), low-density lipoprotein cholesterol (LDL-C), and levels of alanine amino transferase (ALT), aspartate amino transferase (AST), gamma glutamyl transferase (GGT) were determined by automated enzymatic methods and commercial kits (Pars Azmoon, Iran).

Circulating level of FAM19A5 was measured with an enzyme linked immunosorbent assay (ELISA) with a minimum detectable concentration of 15 pg/ml, Intra assay CV of < 10% and Inter assay CV of < 12% (RayBiotech Inc., USA).

### Ultrasonography and elastography

Ultrasound assessment was performed using Accuvix XQ ultrasound unit (Medison, South Korea) equipped with a 3–7 MHz curved-array and a 5–12 MHz linear-array transducer for the evaluation of liver, abdominal fat and carotid arteries as previously described [27]. Ultrasonographic scoring was used to determine fatty liver with specificity of 100% and sensitivity of 91.7%. In this protocol, ultrasonography results included vascular blurring (score 0 to 1), hepatorenal echo contrast and/or liver brightness (score 0 to 3) and deep attenuation (score 0 to 2). A total score of at least 2 was needed for the diagnosis of NAFLD [27].

Visceral Adipose Tissue thickness (VAT) was measured between the anterior wall of the aorta and the internal face of the rectus abdominis muscle perpendicular to the aorta. Ultrasonographic measurements have been shown to have strong correlations with the visceral fat area measured by the computed tomography [27].

Carotid Intima-Media Thickness (cIMT) was assessed as the distance between the lumen–intima interface and the media– adventitia interface, measured at its thickest point on the distal (far) wall of the common carotid arteries, 1.5–2 cm proximal to the carotid bulb. The average of right and left sides was used for cIMT analysis [27].

LS was measured by transient elastography using the FibroScan 502 machine (EchoSense, Paris, France, 5 MHz). According to the manufacturer’s guidelines the M probe was used for the subjects with thoracic perimeter less than 110 cm and the XL probe for 110 cm and above. At least 10 measurements were done for each patient and the median value was recorded. Values were considered valid if the inter-quartile range (IQR) was less than 30% of the median reading [27].

### Statistical analyses

The sample size was calculated based on data from a previous study about circulating levels of FAM19A5 in the subjects with and without diabetes [9]. We calculated that 32 subjects in each group would provide 90% power to achieve a difference of 80% in the circulating levels of FAM19A5 between the studied groups assuming a 2-sided t-test with alpha of 0.05.

The Shapiro-Wilk test was applied to test the normal distribution of values. Non-pairwise comparisons of the concentrations of normally and non-normally distributed variables were performed between the two groups with the students t test and nonparametric Mann–Whitney test respectively. Binary logistic regression models were calculated to identify independent risk factors. Associations between the concentrations of FAM19A5 and other variables of interest were tested with the use of Pearson correlation coefficients after appropriate log-normalizations of the concentration values. Additionally, multivariate linear regressions with the stepwise variable selection were used to test for significant relations in continuous data with adjustment for possible confounders. We used two-tailed hypothesis tests and *P*-values < 0.05 were considered significant.

## Results

The clinical and biochemical characteristics of the study subjects were summarized in Table 1. There were no significant differences between the two groups in terms of age. In the NAFLD group, BMI, waist circumference, FBG, TG, GFR, ALT, AST, GGT and LS were significantly higher than the control group. Whereas HDL-C, LDL-C, TC, creatinine, urea and cIMT were comparable in two groups.

**Table 1:** Baseline clinical characteristics of study participants

	Control	NAFLD	P-value
Age (years)	59.0 (53.5–61.0)	56.0 (54.0–62.0)	0.638
BMI (kg/m2)	25.40 ± 2.92	29.72 ± 2.98	< 0.001
WC (cm)	93.16 ± 9.15	103.81 ± 8.99	< 0.001
TC (mg/dl)	200.71 ± 41.04	215 ± 38.70	0.120
LDL-C (mg/dL)	119.21 ± 36.01	131.16 ± 31.03	0.144
HDL-C (mg/dL)	61 (46–72)	54 (47–64)	0.292
TG (mg/dL)	102 (82–147)	145 (114–180)	0.004
Creatinine (mg/dL)	1.30 ± 0.25	1.35 ± 0.23	0.440
GFR (mL/min)	63.93 ± 17.09	72.79 ± 14.56	0.023
Urea (mg/dL)	27.82 (25.68–33.17)	32.10 (27.82–34.24)	0.053
FBG (mg/dL)	92.72 ± 7.66	101.80 ± 11.59	< 0.001
Visceral fat	43.09 ± 17.24	70.11 ± 18.57	< 0.001
AST (U/L)	20 (17–23)	23 (18–33)	0.031
ALT U/L)	16 (14–23)	31 (22–42)	< 0.001
GGT (U/L)	22.27 (17.60–30.50)	29.21 (23.74–44.22)	0.002
LS (kPa)	3.80 (3.30–4.30)	5.10 (4.50–6.60)	< 0.001
cIMT (mm)	0.79 (0.71 0.85)	0.8 (0.72–1.00)	0.154

The data are expressed as mean ± standard deviation (SD) or median and (interquartile range). BMI: Body mass index; WC: waist circumference; TC: Total cholesterol; LDL-C: Low density lipoprotein cholesterol; HDL-C: High density lipoprotein cholesterol; TG: Triglycerides; AST; aspartate amino transferase; ALT: alanine amino transferase; GGT: gamma glutamyl transferase; LS: liver stiffness; cIMT: Carotid artery intima-media thickness. Independent Student’s t-test and Mann-Whitney U test were used to determine the differences of variables between two groups. *p* < 0.05 was considered statistically significant

**Fig. 1:**
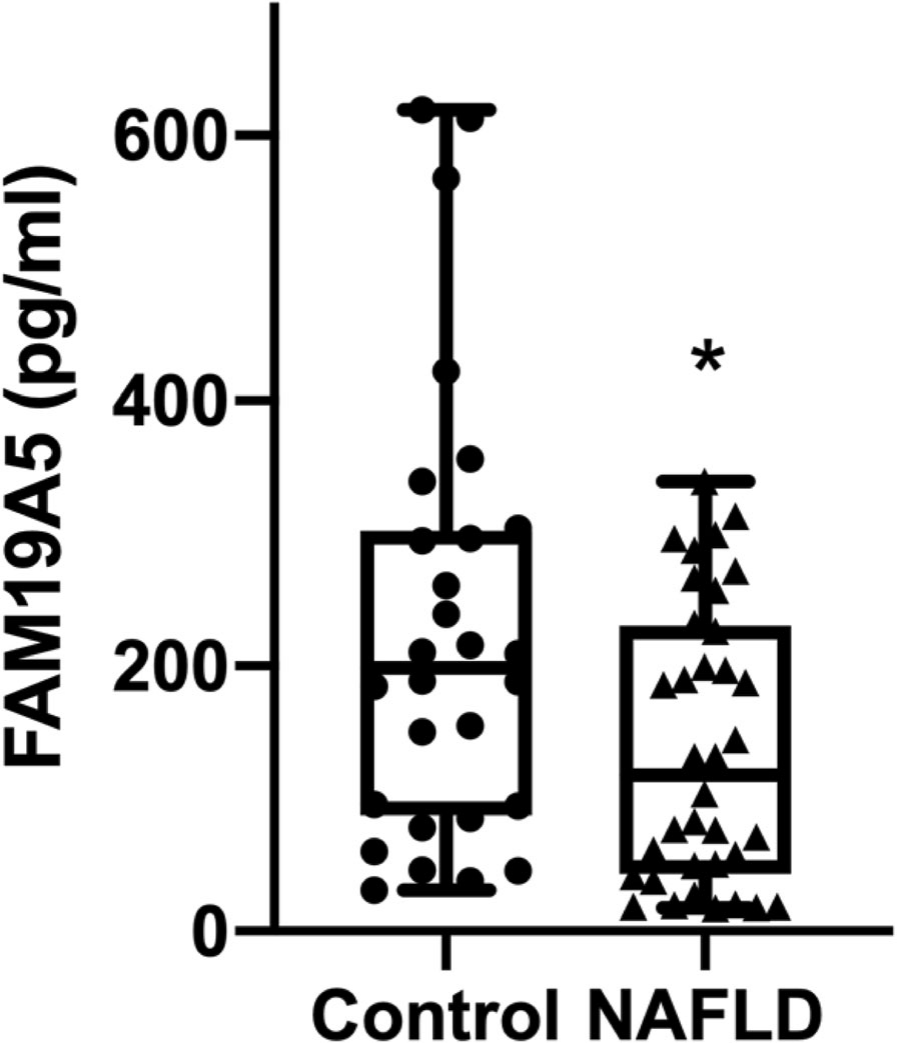
Comparison of plasma levels of FAM19A5 between control and NAFLD groups. Concentration of FAM19A5 in plasma measured by ELISA; differences between the groups were analyzed with Mann-Whitney U; *indicates *p* < 0.05

The plasma FAM19A5 concentration was significantly lower in NAFLD group compared to control group (Fig. 1).

Associations between FAM19A5 plasma levels and presence of NAFLD were assessed using binary logistic regression. FAM19A5 appeared to be negatively associated with presence of NAFLD (*P* = 0.019). This association remained significant after adjustment for age (P = 0.019). It disappeared after adjustment for BMI (*P* = 0.062). But the association of FAM19A5 with NAFLD remained significant after adjustment for the visceral fat (*P* = 0.025) (Table 2).

**Table 2:** The logistic regression model for risk factors of NAFLD

	OR (95% CI)	*P*-value
	Unadjusted	
FAM19A5	0.995 (0.991–0.999)	0.019
	Adjusted for age	
FAM19A5	0.995 (0.991–0.999)	0.019
	Adjusted for BMI	
FAM19A5	0.995 (0.990–1.000)	0.062
	Adjusted for visceral fat	
FAM19A5	0.993 (0.988–0.999)	0.025

Data are presented as odds ratios (ORs) with 95% confidence intervals (CIs) per 1 SD increase in FAM19A5 concentrations. BMI: Body mass index. *p* < 0.05 was considered statistically significant

**Fig. 2:**
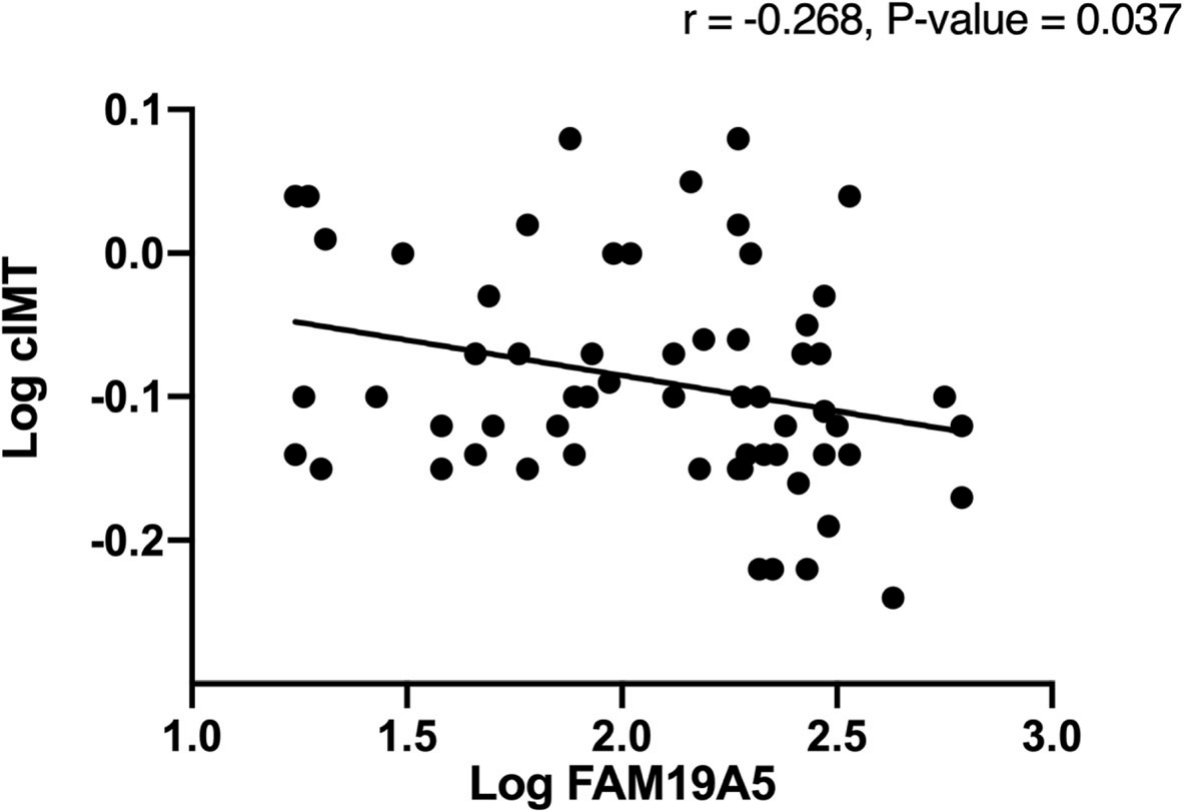
Correlation between plasma level of FAM19A5 and Carotid intima-media thickness (cIMT). Pearson correlation analysis was utilized to investigate the association of the extent of carotid atherosclerosis with FAM19A5 level. Log of Carotid intima-media thickness (cIMT) plotted against log of FAM19A5

The associations between circulating FAM19A5 levels and clinical and biochemical variables were assessed and the data are summarized in Table 2. Plasma FAM19A5 level showed a significant negative correlation with serum levels of liver enzymes including ALT (r = − 0.355, *P* = 0.004) and AST (r = − 0.254, *P* = 0.043) and a significant positive correlation with HDL-C (r = 0.281, *P* = 0.025). There was also significant negative correlation between plasma level of FAM19A5 and liver stiffness (LS) (r = − 0.294, *P* = 0.021). Furthermore, significant negative correlations were found between FAM19A5 levels and BMI (r = − 0.285, *P* = 0.024) as well as FAM19A5 and visceral fat (r = − 0.317, *P* = 0.011). (Table 3). There was also a significant negative correlation between cIMT and FAM19A5 (r = − 0.268, *P* = 0.037) (Fig. 2).

**Table 3:** Pearson correlation coefficients between FAM19A5 and metabolic and anthropometric parameters

Parameter	r	*P*-value
BMI	−0.285	0.024
WC	−0.238	0.06
Visceral fat	− 0.317	0.011
TC	0.063	0.621
Log HDL-C	0.281	0.025
LDL-C	−0.003	0.979
FBG	−0.181	0.151
Log TG	−0.123	0.334
Log GGT	0.006	0.966
Log ALT	−0.355	0.004
Log AST	−0.254	0.043
Log LS	−0.294	0.021

*BMI* body mass index, *WC* waist circumference, *TC* total cholesterol, *LDL-C* low density lipoprotein cholesterol, *HDL-C* high density lipoprotein cholesterol, *TG* triglycerides, *AST* aspartate amino transferase, *ALT* alanine amino transferase, *GGT* gamma glutamyl transferase, *LS* liver stiffness

*p* < 0.05 was considered statistically significant

Stepwise multiple linear regression analysis was performed to determine whether obesity (BMI, visceral fat), fatty liver (ALT, AST, LS) and atherosclerosis (cIMT) indices influenced FAM19A5 levels. ALT (*P* = 0.003) and cIMT (P = 0.024) were found to be the only significant determinants of FAM19A5 levels with the model explaining 20% of the variance in levels of FAM19A5 (log FAM19A5 = 2.887–0.679[log ALT]- 1.539 [log cIMT]) (Table 3).

## Discussion

In the current study, we found lower circulating FAM19A5 concentrations in NAFLD group compared to the control group, suggesting a role of this adipokine in NAFLD risk. To our knowledge, this is the first study ever to report the association of FAM19A5 circulating level with NAFLD.

Several studies have shown association of FAM19A5 gene with neurological and/or psychiatric diseases [22–25, 28, 29]. FAM19A5 has been linked to inflammation, differentiation and cognition [22, 24, 26, 29, 30]. But few studies have addressed the role of FAM19A5 in cardiometabolic disorders. Recently, Wang et al. identified FAM19A5 as a new secretory protein which is highly expressed in adipose tissue [8]. Having analyzed the expression of FAM19A5 in multiple human tissues, they observed FAM19A4 expressed in the adipose tissue even more than the brain [8]. They compared FAM19A5 expression in the epididymal adipose tissues of non-obese and obese mice and found reduced expression of FAM19A5 in both high-fat diet-induced obese and leptin receptor- deficient (db/db) mice compared to the non-obese ones [8]. These results are in agreement with our findings which showed lower circulating levels of FAM19A5 in NAFLD. Moreover, an in vitro study showed that TNF-α-induced inflammation in adipocytes decreased expression of FAM19A5 suggesting that a pro-inflammatory condition as it observed in NAFLD might cause downregulation of FAM19A5 [31, 32]. However, Lee et al. reported higher serum levels of FAM19A5 in diabetic patients compared to non-diabetic subjects [9]. Due to the previously reported protective roles of FAM19A5 in atherosclerosis, obesity and inflammation, we assume that lower levels of FAM19A5 in our study might suggest its protective role in the NAFLD pathogenesis [8, 31, 32]. The clinical relevance of this hypothesis is indirectly supported by the other studies showed decreased levels of other protective adipokines in the NAFLD patients [4, 6, 33]. However, we cannot rule out the possible influence of the race-ethnic difference between study populations. A previous study showed significant race-ethnic differences in circulating levels of other adipokines [34].

In the present study, circulating levels of FAM19A5 was negatively correlated with BMI and the visceral adipose tissue. This finding is in good agreement with the results from Wong et al. which showed negative correlation between FAM19A5 mRNA level of the epididymal adipose tissue and body weights in mice [8]. After adjusting for BMI, logistic regression analysis revealed a borderline significant relationship between FAM19A5 and NAFLD. Moreover, after adjusting for the visceral fat, the association of FAM19A5 with NAFLD remined significant. So, although adiposity is an important confounder when considering the interaction between adipokines and NAFLD, it does not seem to attenuate the relationship between FAM19A5 and NAFLD remarkably. Our findings showed negative correlations between FAM19A5 and fatty liver indices including ALT, AST, and LS.

We have also observed a negative correlation between FAM19A5 and cIMT. In line, previous studies have indicated association of other adipokines with cIMT in NAFLD patients [17, 33]. Moreover, several basic and pre-clinical studies demonstrated the vasculoprotective effects of adiponectin in atherosclerotic models. Atherosclerosis is characterized by neointima formation. One of the major processes occurs in the neointima formation is the proliferation and migration of vascular smooth muscle cells into the intimal layer of the artery. Wang et al. consistently showed adipose derived FAM19A5 inhibited vascular smooth muscle cell proliferation [8]. To study the effect of FAM19A5 on neointima formation in the injured arteries in rats, they overexpressed FAM19A5 in balloon-injured carotid arteries and observed neointima formation was significantly repressed in the FAM19A5 overexpressed vessels [8]. In the current study, when we conducted a stepwise regression analysis to identify parameters that were related to FAM19A5 levels, ALT and cIMT were found to be the parameters were associated significantly with circulating levels of FAM19A5 after controlling for BMI and the visceral fat.

Our study has some limitations. Due to the cross-sectional design of our study, clarification of the causality between FAM19A5 and other parameters was inherently limited. The sample size was relatively small, and further studies with larger sample size are needed to confirm our results. Our study population included only men. Previous studies reported gender differences in circulating levels of adipokines [35, 36]. Although our findings cannot be generalized to the whole population, the confounding effect of sex has been eliminated.

## Conclusions

Here, we demonstrated that the circulating level of FAM19A5 is associated with NAFLD. We also showed a significant and independent correlation between FAM19A5 and cIMT. Our data suggest a protective role of FAM19A5 in NAFLD and subclinical atherosclerosis in patients with NAFLD who have characteristics of high CVD risk. Longitudinal studies are also required to confirm whether lower plasma FAM19A5 levels can potentiate the NAFLD patients to atherosclerosis.

## Data Availability

The datasets during and/or analyzed during the current study available from the corresponding author on reasonable request.

## Abbreviations

NAFLD: Nonalcoholic fatty liver disease
CIMT: Carotid artery intima-media thickness
BMI: Body mass index
ALT: Alanine amino transferase
AST: Aspartate amino transferase
LS: Liver stiffness
FAM19A5: Family with sequence similarity 19 (chemokine (C-C motif)-like) member A5
FBG: Fasting blood glucose
TC: Total cholesterol
TG: Triglycerides
HDLC: High-density lipoprotein cholesterol
LDL-C: Low-density lipoprotein cholesterol
GGT: Gamma glutamyl transferase
